# Implementing Accurx for Total Triage Enhancing Care Navigation and Patient Experience

**DOI:** 10.7759/cureus.78964

**Published:** 2025-02-13

**Authors:** Rahul Mittal, Ajith Kumar Kannan, Rajnish Mohindroo, Chakri Movva, Lai Zhang, Salma Reehana, Shankar Srinivasan

**Affiliations:** 1 Health Informatics, Rutgers University, New Brunswick, USA; 2 General Practice, Health and Beyond Partnership, Wolverhampton, GBR; 3 Health Informatics, Health and Beyond Partnership, Wolverhampton, GBR

**Keywords:** ehr analysis, electronic health record (ehr), emergency informatics, informatics, patient triage

## Abstract

Introduction

Total triage is a transformative approach to healthcare delivery that uses digital platforms such as Accurx to replace traditional appointment scheduling with a remote-first system. Unlike conventional methods, total triage involves an initial remote assessment - via online forms, calls, or video consultations - to prioritize cases based on urgency and allocate resources more effectively. This system addresses challenges such as long wait times, excessive call volumes, and clinician burnout while improving accessibility and ensuring timely, patient-centered care. This research focuses on the challenges of implementation, particularly patterns of use related to call wait times and patient and staff experiences with the Accurx platform.

Methods

This retrospective study analyzed routinely collected data from the Bilston Urban Village (BUV) Medical Center between August 2023 and September 2024, focusing on the implementation of the Accurx total triage platform. A mixed-methods approach was employed to provide a comprehensive evaluation of its impact. Quantitative data were collected on call wait time metrics to assess system efficiency and identify patterns of use. Qualitative data were gathered through a patient satisfaction survey, capturing insights into patients’ experiences. Additionally, staff feedback was taken into account to further understand perceived challenges and areas for improvement. Together, these data sources enabled an in-depth analysis of the platform's implementation phase.

Results

The implementation of the Accurx total triage platform at BUV Medical Center resulted in a 35% reduction in average call wait times compared to pre-implementation levels across all patient demographics, highlighting its efficiency in managing patient demand. Quantitative analysis showed a consistent decrease in wait times over the study period, alongside improved resource utilization. Qualitative data from anonymous patient satisfaction surveys indicated that 58% of respondents found the system easy to use and effective in addressing their healthcare needs. However, 14% reported challenges, including difficulties navigating the platform and delays in responses during peak times. Staff feedback reflected these findings, with the majority emphasizing the platform’s ability to streamline triage and reduce administrative burden while identifying challenges such as the need for better support during high-demand periods. These findings contribute to addressing the broader NHS challenges of managing call volumes and underscore the platform's overall effectiveness while highlighting specific areas for improvement, particularly in accessibility and responsiveness.

Conclusion

The Accurx total triage platform improved efficiency and patient and staff satisfaction at BUV Medical Center, particularly by reducing call wait times and streamlining access to care. Despite these successes, challenges such as navigation difficulties highlight areas for enhancement. Addressing these barriers and optimizing the system’s functionality will be crucial for sustaining its long-term impact. Overall, the study underscores the potential of digital triage systems to transform healthcare delivery by improving access, resource utilization, and patient experiences.

## Introduction

The National Health Service (NHS) is currently grappling with a complex crisis driven by rising demand due to an aging population and the increasing prevalence of chronic conditions. This challenge is compounded by a 4%-5% reduction in full-time equivalent (FTE) general practitioners (GPs), leading to rising consultation numbers and placing immense strain on the remaining workforce, which is increasingly vulnerable to burnout and threatening the sustainability of primary care services [1,2]. As of November 2024, the total number of FTE GPs has risen by 10.7% since December 2019, reaching 38,216. This figure includes 10,077 GP trainees - doctors in specialty training to become GPs - and 28,139 fully qualified GPs, a category that also includes locum GPs, who provide temporary cover for practices to address short-term staffing shortages. However, the number of fully qualified FTE GPs has shown only a marginal increase during this period and has declined by 4.17% since 2015 [3].

Demand for general practice in the United Kingdom (UK) has reached unprecedented levels. In 2023, 356 million appointments were delivered - a 14% rise compared to 2019 - with the average number of patients per GP growing to 2,294 by April 2024, a 7.2% increase over five years [3]. In April 2024 alone, 30.7 million consultations were conducted, including 45% as same-day appointments, representing a 3.3 million increase compared to April 2019. Face-to-face consultations comprised 65% of all appointments, and GP-led appointments rose by 1.1 million to 13.7 million [3]. This surge in demand has placed significant strain on primary care services, leading to longer wait times for both elective and urgent care and increasing pressures on emergency departments. Digital solutions, such as online triage systems and appointment scheduling platforms, aim to improve the efficiency of scheduling while enhancing patient care delivery by prioritizing cases based on urgency. This enables clinicians to focus on the most critical patients. Additionally, these tools streamline administrative workflows, reducing the time spent on non-clinical tasks, and offer patients accessible self-service options for initial consultations, minimizing unnecessary clinic visits [4]. Furthermore, these platforms can support integrated care approaches by improving communication and data sharing among healthcare providers, ultimately leading to more coordinated and personalized care [5]. These measures are essential to alleviate the burden on general practitioners and ensure timely and effective care.

The NHS is also facing significant workforce challenges, particularly in GP. While specific data on front office or scheduling staff shortages is limited, broader staffing issues highlight the strain on administrative roles [6]. The overall workforce pressures within general practice suggest that administrative staff are similarly affected. Implementing digital solutions, such as automated scheduling systems, can help alleviate some of this burden by streamlining appointment management and reducing manual tasks [7,8]. Research indicates that such technologies can lead to increased job satisfaction and reduced stress among healthcare staff, enhancing both efficiency and well-being [5].

The 2024 Darzi Report: strategic recommendations for healthcare modernization 

The 2024 Darzi Report presents a series of strategic recommendations aimed at modernizing healthcare infrastructure, strengthening workforce retention, and expanding digital solutions [9]. A key focus is on integrated care systems, which streamline patient pathways, enhance operational efficiency, and address the backlog in elective care. By fostering a more coordinated approach to service delivery, these systems can alleviate systemic pressures and improve patient outcomes [10,11].

The report emphasizes the urgent need to modernize primary care infrastructure to meet growing demands and reduce strain on GPs. A central challenge facing primary care is the overwhelming patient load, which limits the number of patients each GP can effectively assess and treat within a given time frame [12]. To address these issues, workforce retention strategies - including enhanced training programs and structured support systems - are proposed to mitigate the GP shortfall and build a more resilient healthcare workforce.

These recommendations reinforce the necessity of integrating digital tools, such as total triage systems, to improve access, optimize resource utilization, and support overburdened GPs in managing patient demand more effectively. By streamlining administrative processes, prioritizing urgent cases, and reducing unnecessary consultations, digital solutions can help alleviate pressures on primary care [13]. As these technologies continue to evolve, they have the potential to transform healthcare delivery, ensuring a more sustainable, efficient, and equitable system. 

The NHS’s digital transformation: strategy and implementation 

The NHS has increasingly prioritized digital transformation as a cornerstone strategy to modernize healthcare delivery, improve patient access, and streamline service provision. Digital strategies within the NHS are guided by policies aimed at integrating technology across all aspects of healthcare operations [14]. A key example is the NHS long-term plan, which emphasizes expanding online services, digital consultation tools, and AI-based triage systems to enhance the patient experience and improve operational efficiency [15].

The impact of COVID-19 on digital adoption 

The COVID-19 pandemic significantly accelerated the adoption of digital triage systems in primary care, as healthcare providers swiftly integrated these tools to facilitate remote consultations and minimize infection risks. This surge in demand spurred rapid advancements in telemedicine platforms, with existing providers expanding their capabilities to meet emerging healthcare challenges [16]. The UK healthcare system was well-positioned for this transition, as video and e-consultation services had been growing since 2018. Supported by Clinical Commissioning Groups (CCGs) in England and government-funded initiatives in Scotland and Wales, platforms such as Accurx and Anywhere Consult were quickly deployed [17]. By early 2020, Accurx’s chain short message service (SMS), which enables GPs to send secure messages directly from patient records, was already in use at half of England’s GP practices. In response to the urgent need for remote care solutions, many technology companies offered their services at minimal or no cost by March 2020 [18].

However, the shift to digital healthcare exposed disparities in access, particularly among patient groups with limited digital literacy or inadequate technology [19]. These challenges underscored the need for an inclusive approach that ensures equitable healthcare delivery. Hybrid models, which combine digital tools with in-person consultations, present a viable solution by maintaining efficiency while addressing accessibility concerns [18]. Such an approach would enable healthcare providers to leverage technological advancements without exacerbating existing inequalities in service delivery.

Total triage

Total triage is an essential workflow in general practice, designed to collect patient information and assess the urgency and nature of care required before scheduling appointments [20]. This process ensures efficient resource allocation, prioritization of urgent cases, and enhanced patient access to appropriate care. The introduction of digital triage tools, such as online forms, AI-driven algorithms, and telehealth platforms, has significantly enhanced traditional triage systems, making them faster and more adaptable to meet increasing healthcare demands and workforce pressures [21].

Pioneered by platforms such as askmyGP in 2018, digital triage models have revolutionized care delivery by adopting a digital-first approach to assess patient needs, streamline response times, and reduce unnecessary visits to emergency departments. Supported by NHS England, tools such as AlldayDr Remote Online Consultation, Anywhere Consult by EMIS Health, Q doctor, Evergreen Life, SystmOne by TPP, FootFall by Silicon Practice, RIVIAM Secure Video Services, eConsult, PATCHS, Klinik Healthcare Solutions, and Smart Triage by Rapid Health have become integral components of NHS general practice workflows [16,22-28]. These platforms enhance operational efficiency, alleviate pressures on healthcare providers, and enhance overall patient satisfaction by facilitating timely and effective care delivery. As patient demand continues to rise, the transition from traditional to digital triage highlights the necessity for innovation in healthcare workflows. This evolution ensures equitable access, improved resource management, and sustainable practices for the future [20,29].

Accurx implementation and evolution 

Initial Deployment 

Accurx, a health tech start-up, played a pivotal role in transforming primary care during the COVID-19 pandemic. Initially launched in March 2020, the platform was made freely available for one year to all primary care practices, facilitating widespread adoption at a time of critical need [18]. Originally designed for secure SMS communication between GPs and patients, Accurx quickly expanded its capabilities in response to the pandemic. A video consultation feature was introduced within weeks, allowing clinicians to conduct remote appointments efficiently. By the end of March 2020, more than 80% of UK GP practices had adopted the platform, demonstrating its rapid integration into primary care workflows [30].

A key factor in Accurx’s success was its seamless integration with widely used electronic patient record (EPR) systems, such as EMIS and SystmOne. This interoperability enabled GPs to securely communicate with patients, send video consultation links, share documents, and provide NHS resources - all within a single interface [16]. These functionalities improved accessibility and streamlined patient interactions, reducing administrative burden and enhancing the efficiency of remote consultations. 

The Wolverhampton region emerged as a strong case study for Accurx’s impact. Primary care practices in the area reported overwhelmingly positive feedback regarding the platform’s ease of use and effectiveness. Recognizing its value, the NHS Black Country Integrated Care Board (ICB) opted to fund Accurx licensing fees for local practices, ensuring its continued availability and adoption. This strategic investment solidified Accurx’s role in modernizing patient inquiries, prioritizing care delivery, and supporting remote healthcare solutions across the region. 

Feature expansion and adoption 

By May 2023, Accurx introduced additional features aimed at improving operational efficiency, including advanced scheduling and triage tools. Health and Beyond (H&B), based in Wolverhampton, became the first adopter of these new features, deciding in August 2023 and implementing them the following month. Before the introduction of total triage, staff had to manually access EMIS, call patients, and send text messages. This process was time-intensive and less efficient, highlighting the need for automated solutions to streamline workflows. 

Training and implementation challenges 

The initial training for Accurx at Bilston Urban Village (BUV) Medical Center primarily consisted of instructional videos, with no formal training provided during the early stages of implementation. To address emerging challenges, supplementary training sessions were later introduced for reception staff, ensuring they could navigate the system effectively. While this approach enabled a swift rollout, it underscored the necessity of comprehensive training programs to optimize system utilization and streamline workflows. Well-structured training is essential for equipping staff with the confidence and skills needed to integrate digital tools seamlessly into daily operations.

Patient engagement and communication strategy 

A structured patient engagement strategy was developed to facilitate the transition to Accurx total triage at BUV Medical Center. While no open days were held, the Patient Participation Group (PPG) played a critical role in enhancing patient awareness and promoting system adoption. The PPG collaborated with the practice to develop effective communication strategies, assisting with patient education initiatives to ease the transition.

Pre-implementation patient outreach 

In the four weeks leading up to the system’s launch on September 27, 2023, targeted text messages were sent via Accurx to all registered patients. These messages provided clear, step-by-step guidance on the new triage process:

Four and two weeks before launch: Patients received introductory messages explaining the online request system and how to submit triage requests via the BUV Medical Center website. 

One week before launch: A follow-up notification was sent, including an explainer video demonstrating the system’s functionality. 

Final reminder before launch: A last message reinforced key information and provided additional instructional materials, including YouTube videos on the triage process. 

In-person support and information dissemination 

Beyond digital outreach, the PPG provided hands-on support to assist patients and staff during the transition. PPG members guided patients through the new system, addressing concerns and ensuring accessibility for all users. Additionally, printed informational materials were distributed to further enhance patient understanding and encourage engagement. 

By combining digital communication with direct patient support, BUV Medical Center aimed to ensure a smooth transition, enhance accessibility, and minimize disruptions during the implementation of Accurx total triage. 

Accurx online triage platform

The Accurx workflow begins with the patient initiating contact through various channels, such as walk-ins, phone calls, or online forms available via the NHS app. The reception team assists the patient by completing a triage form, either with or on behalf of the patient, to collect essential preliminary information.

These requests are subsequently routed to an inbox, where they are either resolved immediately or assigned to an appropriate colleague for further action. Follow-up actions, tailored to the patient’s needs may include providing a self-booking appointment link, distributing a patient questionnaire, or initiating a two-way conversation to obtain additional details. This streamlined process ensures patients receive timely care, whether through local healthcare services, self-care guidance, or direct clinical interventions.

Although several articles have explored Accurx’s role as a communication platform, there is limited research specifically addressing the impact of its total triage system on call-handling efficiency [31,32]. By examining total call volume and handling efficiency before and after the implementation of Accurx at BUV Medical Center, this study adds valuable insights to the growing discussion on digital triage systems. It also highlights how platforms such as Accurx support the NHS’s dual goals of enhancing the patient experience and streamlining operational processes [33].

Despite its early success and rapid adoption, several critical questions about Accurx remain unanswered. For instance, its long-term influence on healthcare outcomes is unclear. Has the platform consistently improved care quality and promoted health equity, or has it inadvertently widened disparities among vulnerable populations? While clinicians have praised Accurx for its simplicity and efficiency, its broader impact on patient-doctor relationships has yet to be thoroughly examined. Digital consultations may lack the depth of non-verbal communication present in face-to-face interactions, potentially affecting trust and empathy in clinical encounters [34,35]. 

By addressing these gaps in the literature, this study aims to contribute to a more comprehensive understanding of Accurx’s capabilities and limitations, particularly its total triage system, and its implications for primary care.

## Materials and methods

Study design, setting, and duration

This was a retrospective analysis of routinely collected telephone data conducted over 13 months, from August 2023 to September 2024. The research took place at the BUV Medical Center, based in Wolverhampton, UK, with nearly 14,000 patients registered to this practice. The authors did not examine data before the implementation of the Accurx total triage system prior to August 2023 as this was not available in a consistent format.

The study was a collaborative effort conducted by researchers from both Rutgers University, NJ, and the BUV Medical Center. The research question and study framework were conceptualized and designed by authors from Rutgers University, who led the overall study coordination and developed the methodology for data collection and analysis, focusing on helping develop the implementation of the patient and staff satisfaction survey.

Regular virtual meetings were held throughout the study to align objectives, timelines, and responsibilities. A cloud-based platform was utilized for sharing documents, datasets, and iterative drafts of the manuscript to facilitate seamless collaboration. Contributions were integrated and refined collaboratively, ensuring a cohesive and comprehensive analysis.

Study population and selection criteria

The study population comprised patients who accessed healthcare services through the BUV Medical Center between August 2023 and September 2024. Inclusion criteria focused on individuals who utilized the Accurx total triage platform for healthcare appointments during this period. Additionally, healthcare staff involved in managing the triage process were included in the qualitative component to provide insights into system implementation and workflow challenges. This defined population ensured a focused evaluation of the platform's impact on patient and staff experiences.

Sample size

The study included a total of 7,000 patients who utilized the Accurx total triage platform for healthcare access at the BUV Medical Center between August 2023 and September 2024. Quantitative data were derived from all recorded triage interactions, while a subset of 221 patients participated in a satisfaction survey to provide qualitative insights. Additionally, seven healthcare staff members involved in the triage process gave feedback to capture their experiences and perspectives. This sample size ensured a robust evaluation of both patient outcomes and staff workflow dynamics.

Study variables and data analysis

The Accurx total triage system was implemented alongside the X-on Health telephone system at the BUV Medical Center [36]. The X-on Health system is a cloud-based telephony solution specifically designed for healthcare providers to enhance call management and improve patient communication. It integrates seamlessly into healthcare workflows, offering features such as call recording, real-time reporting, and automated call distribution. These functionalities are particularly valuable in managing high call volumes and streamlining patient interactions, making it an essential tool in general practice settings.

The analysis spanned 13 months, from August 7, 2023, to September 23, 2024, with the implementation of Accurx on October 9, 2023, dividing the study into two phases: the Pre-Accurx Period (August 7, 2023-October 8, 2023) and the Post-Accurx Period (October 9, 2023-September 23, 2024). Hourly call data were extracted from the X-on system and supplemented by Accurx usage reports. Key variables included hourly call volume, defined as the total number of calls received during each hour; peak-hour volume, identified as 8:00 AM to 10:00 AM; call response times, focusing on the percentage of calls answered within 5 and 10 minutes; and overall efficiency metrics, such as weekly call volumes and trends before and after Accurx implementation. The analysis was performed using Microsoft Excel (Microsoft® Corp., Redmond, WA).

Qualitative data, including patient and staff feedback, were collected and analyzed to gain a deeper understanding of user experiences. Authors, from Rutgers University, led this component of the study, utilizing thematic coding to patient satisfaction survey and staff feedback. This method allowed for the identification of recurring themes and patterns, providing nuanced insights into the perceived benefits and challenges of the Accurx system. These findings complemented the quantitative analysis, offering a comprehensive evaluation of the system’s impact on care delivery and user satisfaction.

The list of questions asked of the patients is presented in Table 1.

**Table 1:** Custom patient satisfaction questionnaire used at the Bilston Urban Village (BUV) Medical Center

Q. No	Questions
1	How did you come to know about the service?
2	How did you submit the request?
3	How easy was it to submit a triage request?
4	What was the query about?
5	How Satisfied are you with the Service?
6	What would you do if you did not have the option to submit a triage request?
7	Any comments/suggestions?

Ethical consideration

This study involved a retrospective analysis of routinely collected quality performance metrics and patient satisfaction data, for which formal ethical approval was not required. All data were anonymized by the BUV Medical Center to ensure confidentiality, with no identifiable personal information accessed. The study adhered to relevant data protection regulations, including the General Data Protection Regulation (GDPR), to safeguard the privacy and security of the data used.

## Results

Quantitative analysis

Pre-Accurx Call Management Metrics and Challenges 

**Figure 1:**
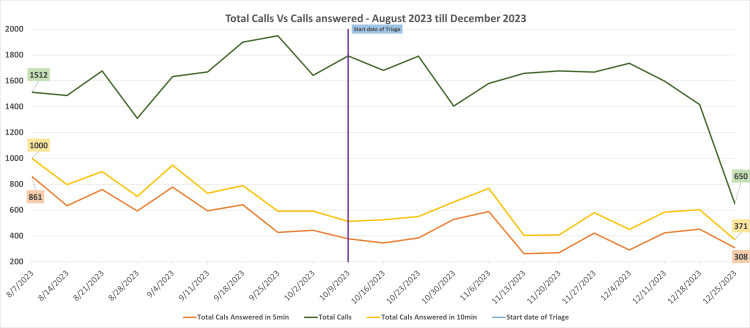
Line graph highlights the BUV Medical Center telephone calls answered before and immediately after the implementation of Accurx from August 7, 2023, to December 25, 2023 The X-axis contains the weekly date range, and the Y-axis denotes the number of calls received and answered.

During the first week of August 2023, the BUV Medical Center managed a total of 1,512 calls in a week, with 56.9% of calls answered within 0-5 minutes and 66.1% answered within 0-10 minutes. Hourly call volumes peaked between 8:00 AM and 10:00 AM, reaching a maximum of 777 calls on September 18, 2023. Prior to the integration of Accurx, call metrics highlighted significant challenges in meeting timely response targets due to high call volumes. On average, only 40% of calls were answered within five minutes, and 49% of calls were answered within 10 minutes (Figure 1).

Post-Accurx Call Management Metrics and Challenges 

The Accurx system was implemented on October 9, 2023, resulting in an initial decline in responsiveness due to transitional challenges, with only 21.0% of calls answered within 5 minutes during the first week. However, by December 2023, call-handling performance steadily improved. The percentage of calls answered within 0-5 minutes increased to 37.6%, and those answered within 0-10 minutes increased to 47.2%, indicating early signs of improved efficiency.

**Figure 2:**
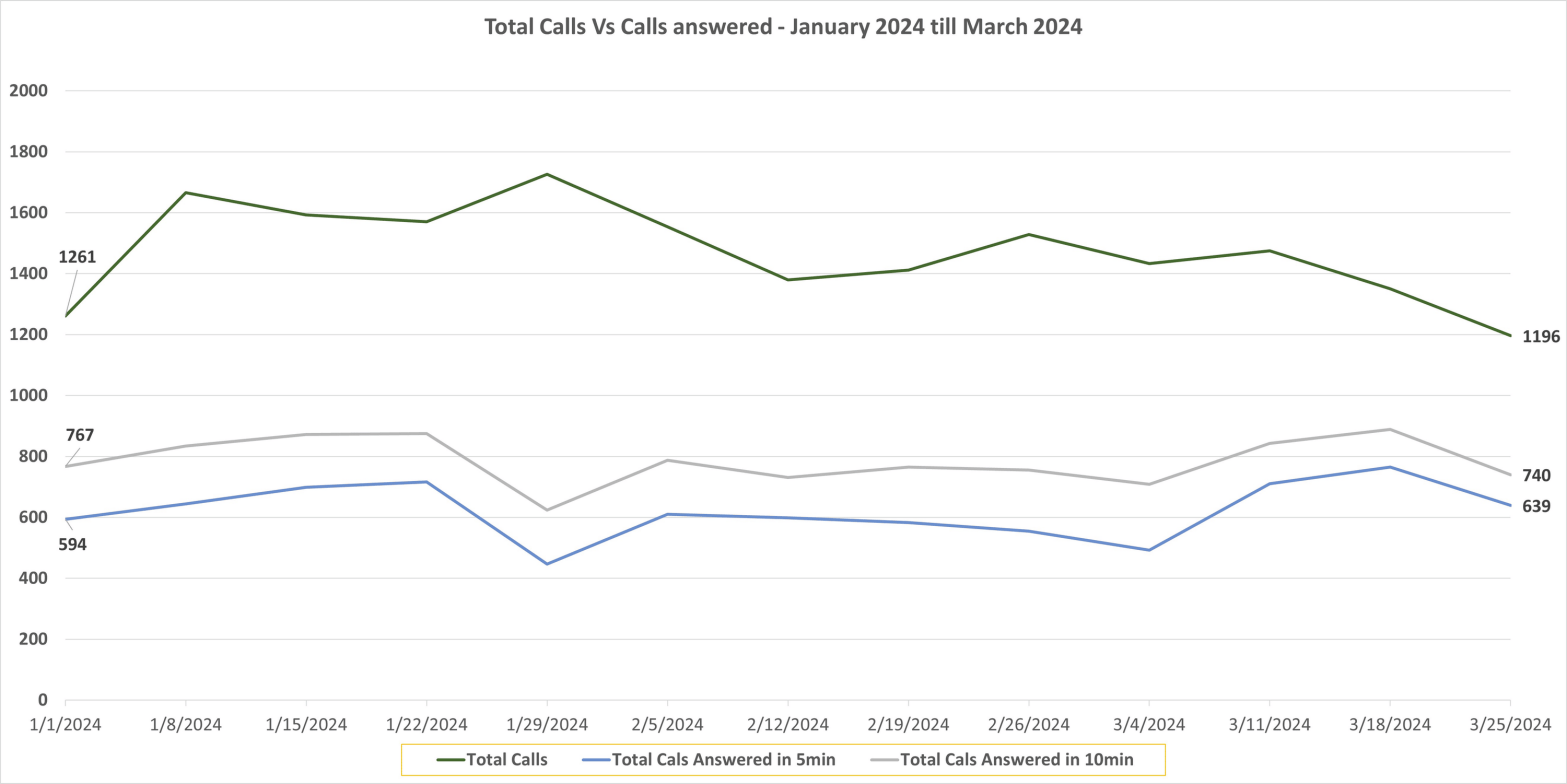
Line graph highlights the BUV Medical Centre telephone calls answered during the period January, 2024, to March 25, 2024 The X-axis contains the weekly date range, and the Y-axis denotes the number of calls received and answered.

Following the transition, the operational efficiency of the BUV Medical Center’s triage system regained effectiveness and began improving. Weekly call volumes stabilized between 1,400 and 1,500, reflecting a slight reduction compared to pre-Accurx levels. The percentage of calls answered within 0-5 minutes steadily increased, reaching 56.7% by mid-March 2024, whereas calls answered within 0-10 minutes improved to 65.9% (Figure 2). By the end of May 2024, total call volume decreased to 916, and the percentages of calls answered within 0-5 mins and 0-10 mins increased significantly to 72.9% and 80.5%, respectively.

During this period, the integration of Accurx’s total triage option contributed to a reduction in direct call volumes as patients increasingly utilized digital platforms for their queries. This trend highlights the effectiveness of the system in alleviating pressure on staff while maintaining high response quality. Seasonal factors, such as reduced call volumes during the holiday season, and staffing dynamics also influenced fluctuations in responsiveness.

**Figure 3:**
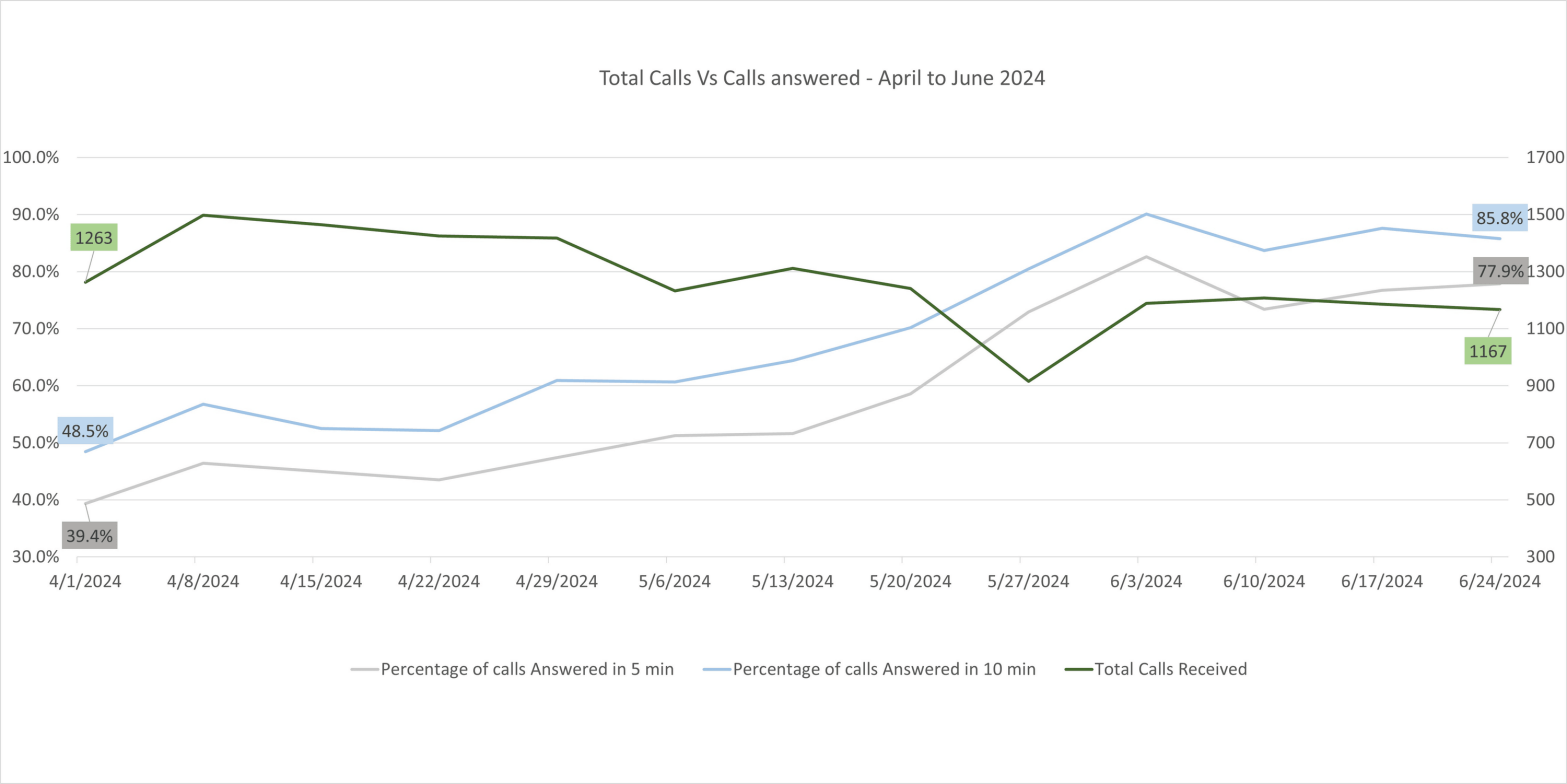
Line graph shows total calls vs calls answered at the BUV Medical Center from April 1, 2024, to June 24, 2024 The X-axis contains the weekly date range, and the Y-axis denotes the percentage of calls received and answered.

From April to June 2024, the performance of the BUV Medical Center's call-handling metrics showed marked improvements (Figure 3). Weekly call volumes averaged around 1,200 to 1,300 calls, with percentages of calls answered within five minutes reaching 72.9% by late May and increasing to 77.9% by early June. Similarly, calls answered within 10 minutes peaked at 85.8% during this period, reflecting steady enhancements in response times.

**Figure 4:**
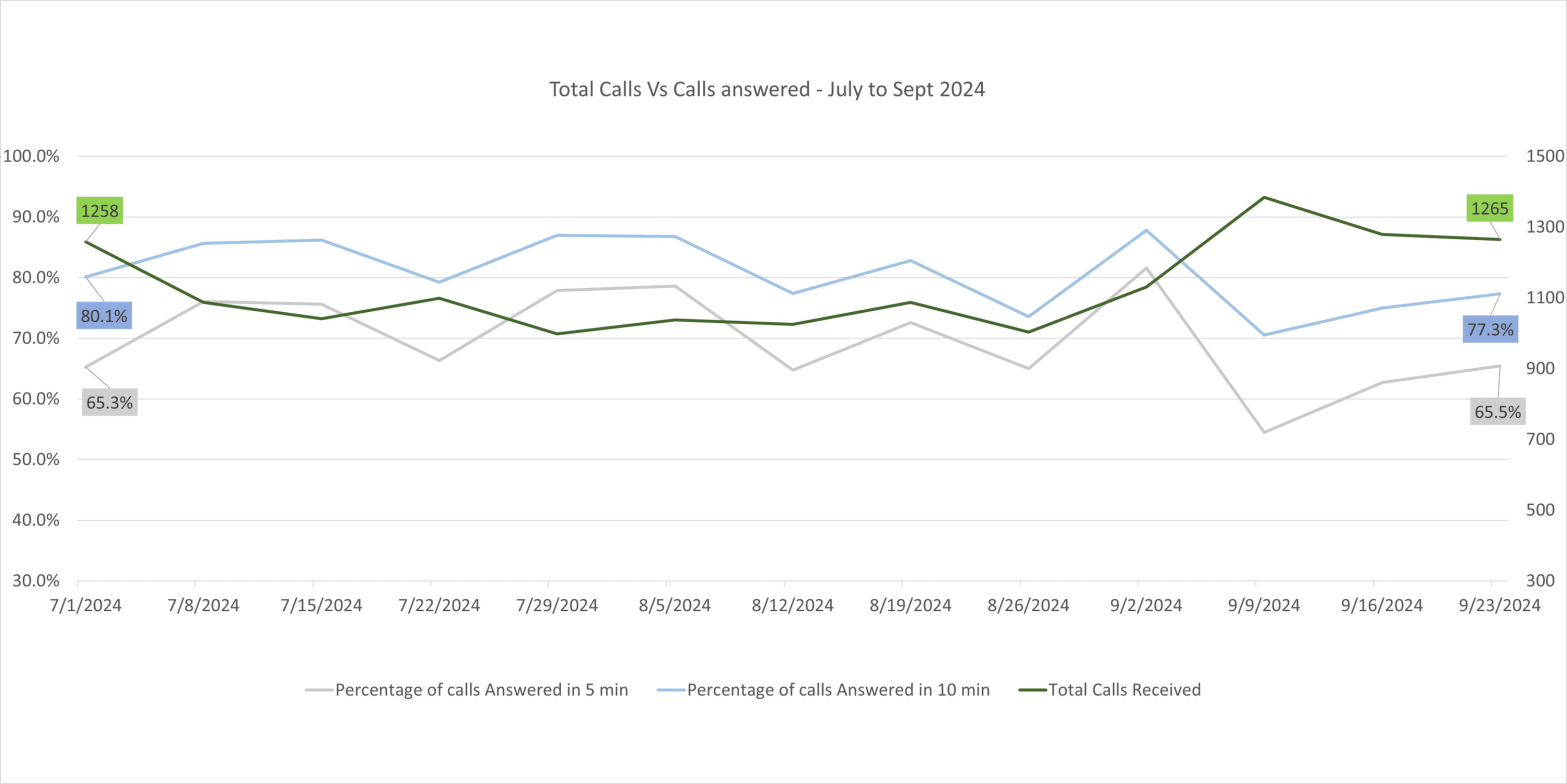
Line graph showing total calls vs calls answered at the BUV Medical Center from July 1, 2024, to September 23, 2024 The X-axis contains the weekly date range, and the Y-axis denotes the percentage of calls received and answered.

Between June and September 2024, the BUV Medical Center maintained its improved performance metrics (Figure 4). Weekly call volumes decreased further, averaging 1,265 calls by September 23, 2024, reflecting the increased adoption of Accurx’s messaging and triage features. On this date, 65.5% of calls were answered within 0-5 minutes, and 77.3% within 0-10 minutes, representing the highest responsiveness achieved during the evaluation period.

The consistent reduction in call volumes, coupled with higher percentages of timely responses, underscores the effectiveness of Accurx in streamlining patient communication. The transition to online triage allowed the BUV Medical Center to address staffing constraints while ensuring timely service delivery. Overall, the system has proven instrumental in enhancing operational efficiency and patient satisfaction.

Patient satisfaction and survey insights 

Since December 2023, the BUV Medical Center has been conducting regular online surveys to assess patient satisfaction and the usability of the Accurx total triage system. The most recent survey, conducted in October 2024, gathered 221 responses, offering valuable insights into patient experiences and areas for improvement.

Ease of use and patient experience 

**Figure 5:**
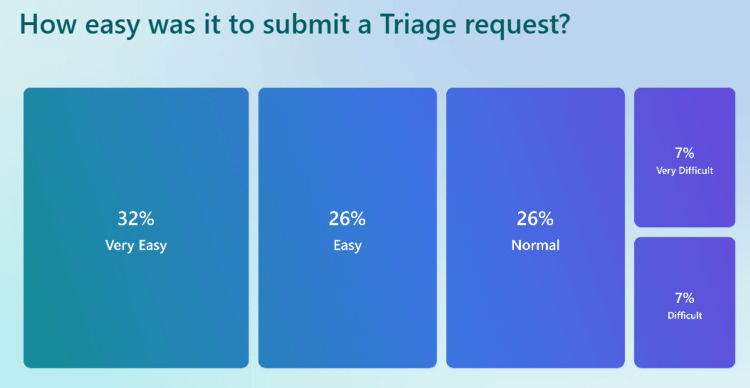
Survey results regarding the ease of use in submitting a total triage request

Regarding the ease of submitting a triage request (Figure 5), 32% of respondents found the process Very Easy, whereas 26% rated it Easy, indicating that a majority (58%) considered the system user-friendly. However, 7% of patients found it Difficult, and another 7% rated it Very Difficult, highlighting accessibility challenges for some users.

**Figure 6:**
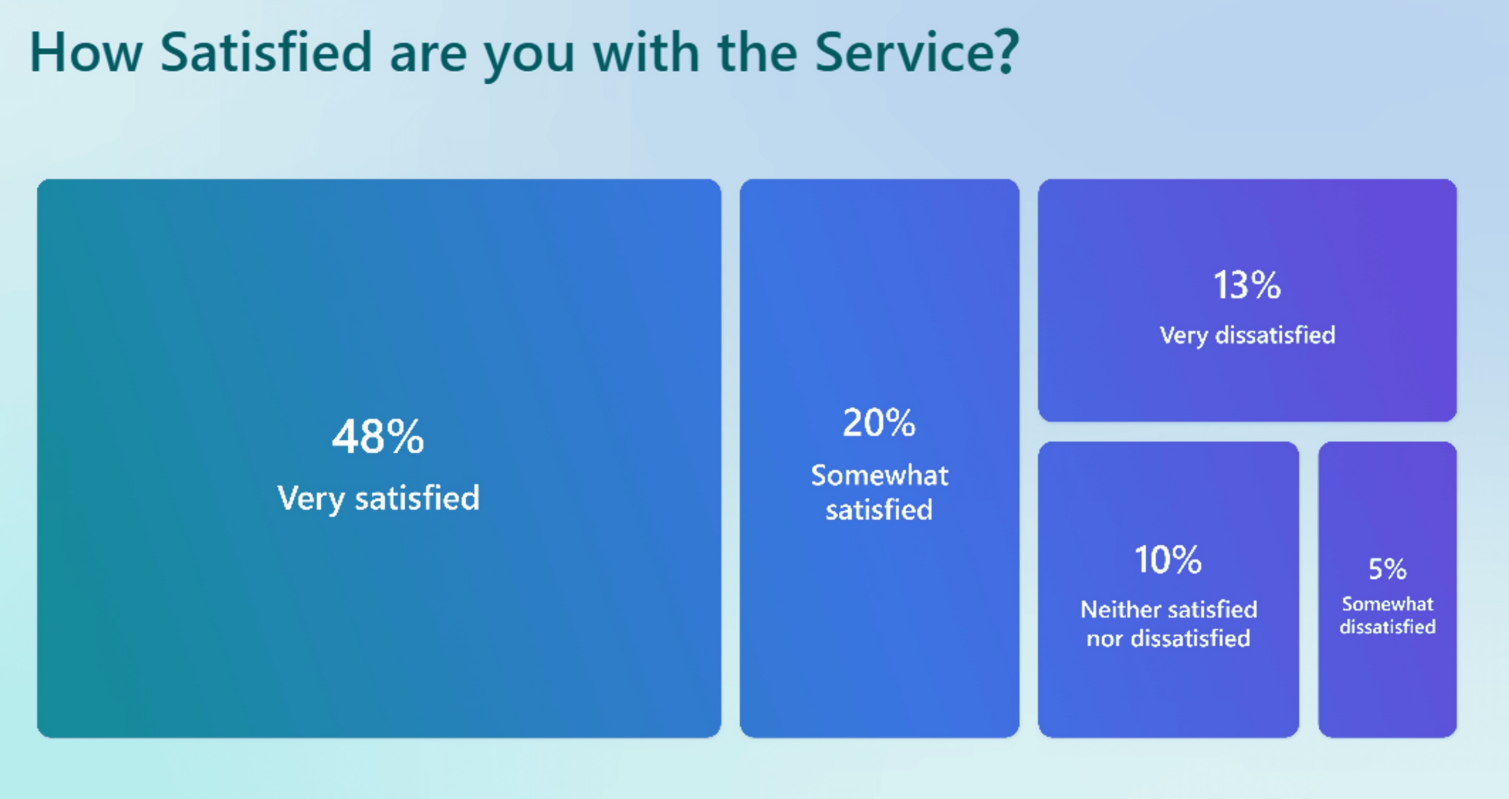
Survey results regarding the overall satisfaction of using the total triage service

In terms of overall satisfaction (Figure 6), 48% of patients reported being Very Satisfied, and 20% were Somewhat Satisfied, resulting in 68% positive feedback. Conversely, 13% of respondents were Very Dissatisfied, and 5% were Somewhat Dissatisfied, suggesting areas requiring further refinement to enhance patient experience.

Patient awareness and use of the triage system 

**Figure 7:**
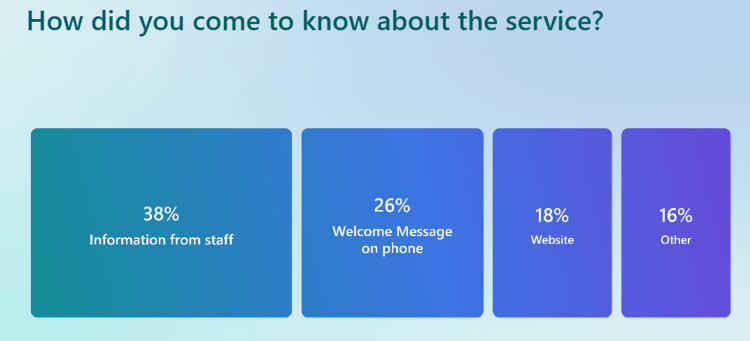
Survey results asking patients how they came to know about the triage system

**Figure 8:**
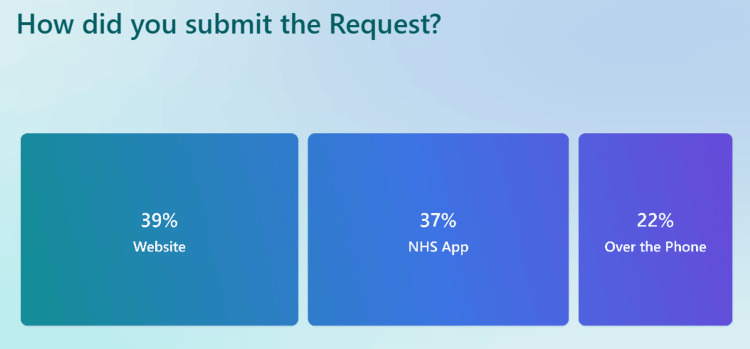
Survey results showing how patients submitted their triage request

When asked how they learned about the triage system (Figure 7), 38% of patients cited BUV Medical Center staff, 26% mentioned the welcome message on the phone, 18% discovered it via the practice website, and 16% through other sources. The preferred method for submitting triage requests also varied (Figure 8), with 39% using the practice website, 37% opting for the NHS app, and 22% choosing to call the practice directly.

Nature of patient queries 

**Figure 9:**
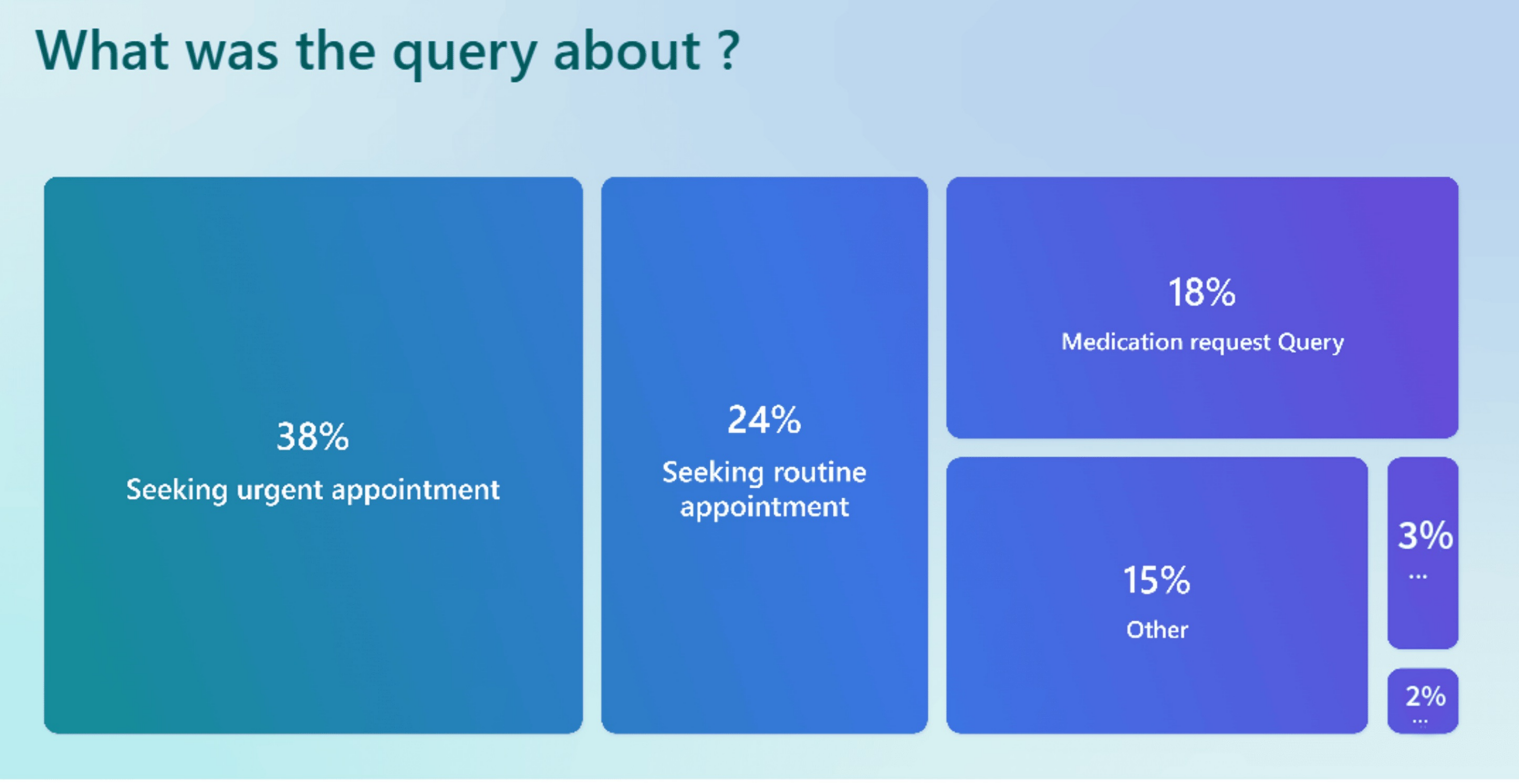
Survey results showing the common reason for using the triage system

The most common reason for using the triage system (Figure 9) was requesting an urgent appointment (38%), followed by routine appointment requests (24%). Additionally, 18% of patients submitted medication-related queries, while 15% selected "Other." A smaller proportion reported queries related to reception and administrative tasks (2%) and medication requests separately (3%).

Patient behavior in the absence of digital triage 

**Figure 10:**
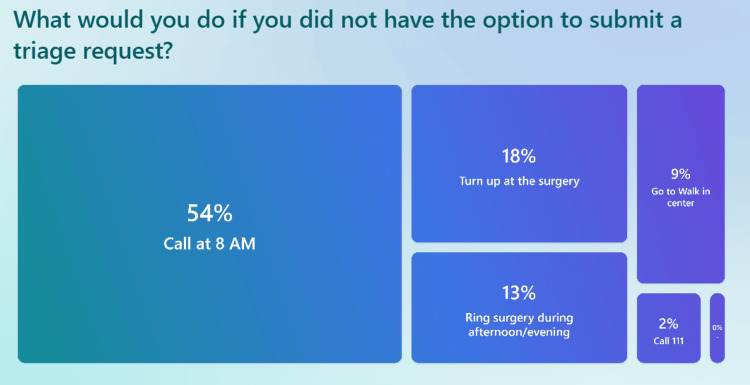
Survey results asking patients what they would do if the triage system were unavailable

When asked what they would do if the triage system were unavailable (Figure 10), 54% of patients stated they would call the practice at 8:00 AM, reflecting the ongoing reliance on early-morning phone calls to secure appointments. This behavior aligns with broader NHS trends, where the "8 AM scramble" persists both online and via phone, as seen in NHS App booking patterns [37,38].

Survey results asking patients what they would do if the triage system were unavailable.

Key takeaways and areas for improvement 

The survey results underscore the benefits of Accurx in improving triage efficiency while identifying areas for refinement. While most patients found the system easy to use, some faced difficulties that needed to be addressed through better accessibility measures. Enhancing patient awareness through more targeted communication and providing additional support for users struggling with digital access could further optimize the triage process. Additionally, offering alternative pathways during peak demand could help alleviate pressure on the system and ensure smoother patient experiences.

Qualitative analysis

Patient Feedback

While many patients appreciate the convenience and efficiency of the Accurx total triage system, challenges remain in ensuring inclusivity, timeliness, and user-friendliness (Table 2). Suggestions for improvement focus on expanding system accessibility, enhancing follow-up communication, and offering alternative appointment-making methods to accommodate diverse patient needs.

**Table 2:** Thematic analysis of patient feedback survey at the BUV Medical Center done on October 2024

Themes	Comments
System Usability and Accessibility	Positive: Many patients appreciated the convenience of the system, especially for repeat prescriptions and avoiding long phone queues. Example: "I think this online service is great! Saves me having to drive to the surgery to submit a repeat prescription request."
	Negative: A significant number of respondents expressed difficulty navigating the system, particularly older adults or those not proficient with technology. Example: "Going online is not easy for the elderly. I'm 74 and sometimes it can be frustrating for me.
Timeliness and Responsiveness	Positive: Some patients found the system efficient for urgent issues, minimizing the need for extended phone hold times. Example: "Very convenient to be able to access help in emergency situations."
	Negative: Others reported delays in responses, even for urgent queries, leading to dissatisfaction. Example: "I did a form for my son as he was really unwell, and I’m still waiting for contact about that."
Preference for Traditional Systems	Many patients expressed a preference for traditional appointment-making methods, citing challenges with digital systems and a desire for human interaction. Example: "I still believe online triage should be scrapped. Much better to speak to a human."
Health Outcomes and Service Quality	Positive: Some patients found the triage system efficient in managing their healthcare needs and minimizing unnecessary visits to the clinic. Example: "I think it's a much fairer system as all forms are triaged rather than being told there are no appointments and to try again tomorrow."
	Negative: Others raised concerns about suboptimal outcomes, such as being directed to pharmacists when a GP consultation was necessary. Example: "I was sent to a pharmacist who couldn't do anything for my son's suspected glandular fever."
System Limitations and Suggestions for Improvement	Patients noted operational limitations, including restricted availability of the system and lack of follow-up on submitted queries. Example: "I really like the service, but I don't like that it's only available for a few hours a day." Suggestions included expanding system hours, improving follow-up communication, and rethinking the process for urgent care. Example: "It would be a better service if a message was sent to the patient in cases where a request was not approved."
Impact on Vulnerable Populations	Feedback highlighted the system's inadequacy for vulnerable groups, such as the elderly, non-tech-savvy individuals, or those with mental health challenges. Example: "The system needs to be better and not addressed to minors, especially those with disabilities."

Staff Feedback

The thematic analysis (Table 3) reveals that staff feedback aligns with key metrics of the system’s success, including enhanced efficiency, reduced stress, and increased patient satisfaction. However, the analysis also underscores the importance of maintaining these improvements and addressing any residual challenges.

**Table 3:** Thematic analysis of staff feedback on Accurx implementation at the BUV Medical Center The frequency count indicates that themes related to efficiency and reduced call volumes were the most commonly mentioned, followed by staff morale and patient satisfaction. This highlights the primary perceived benefits of the Accurx total triage system among staff.

Themes	Comments	Frequency of Mentions (n=7)
Theme 1: Enhanced Efficiency in Patient Triage and Management	Several comments highlighted the improved efficiency of the triage system in managing patient demand and directing them to appropriate services. For instance, one staff member noted that it is "a better way of managing and directing patients to ensure appropriate use of services," while another mentioned, "Patients dealt with same day."	3
Theme 2: Reduced Call Volumes and Wait Times	The reduction in call volumes and waiting times was frequently emphasized as a key benefit. Staff remarked on the significant decrease in phone queues, particularly during peak hours. One comment stated, "Less calls and patients dealt with same day," and another noted, "You don’t have to wait ages in a phone queue."	3
Theme 3: Improved Staff Morale	Staff described a noticeable improvement in morale, attributing it to reduced stress levels and better workflow management. For example, one respondent shared, "Much better staff morale without being stressed with massive call volumes at 8 in the morning."	2
Theme 4: Increased Patient Satisfaction	Staff perceived patients as being more satisfied with the triage system, which positively impacted their own experiences. Comments like "Patients are happier with the system" and "Significantly less complaints, which is great for me" reflect this sentiment.	2
Theme 5: Positive Overall Assessment of the System	Overall, staff expressed a favorable view of the triage system, describing it as "working very well" and a "great service." The overarching tone suggests strong approval of the system's implementation and impact on both patient care and workplace dynamics.	2

## Discussion

The findings of this study highlight the significant impact of the Accurx total triage system in transforming healthcare delivery at the BUV Medical Center. The implementation of this system has demonstrated improvements in call efficiency, reductions in wait times, and increased patient satisfaction. These outcomes underscore the potential of digital triage systems to enhance primary care workflows and improve the patient experience, aligning with similar findings reported in the literature [39,40].

A closer examination of call volume trends reveals the influence of both demographic and seasonal factors. Call volumes exhibited seasonal variations, with a noticeable decrease from mid-December 2023 to mid-January 2024 due to the winter holidays, a period typically associated with reduced healthcare demand. However, by June 2024, call volumes stabilized, averaging approximately 1,250 calls per week. These trends suggest that patient engagement with digital triage systems may fluctuate based on external factors, such as holiday periods and seasonal illness patterns. Moreover, demographic factors, including patient age and digital literacy, likely influenced system adoption. While younger, tech-savvy patients found the online system convenient, older patients or those less familiar with digital technology may have faced challenges, necessitating additional support mechanisms.

Before the implementation of Accurx, the existing X-on Health system identified significant inefficiencies, including high call volumes, extended wait times, and a substantial burden on staff. The introduction of Accurx addressed some of these challenges by optimizing patient prioritization, reducing redundant calls, and streamlining care coordination. The quantitative results demonstrated notable improvements, including a 35% reduction in average call wait times and a significant increase in the proportion of calls answered within targeted response windows. These outcomes highlight the system's ability to enhance operational efficiency and effectively manage patient demand, offering a scalable solution to the challenges of primary care delivery.

Additionally, qualitative feedback from patient satisfaction surveys revealed that 58% of respondents found the system easy to use and effective in addressing their healthcare needs. However, 14% of respondents highlighted challenges, including navigation difficulties and delays during peak times. A thematic analysis of this feedback uncovered key trends. System usability emerged as a significant factor, with many patients appreciating the convenience of avoiding long phone queues. However, some elderly or non-tech-savvy users reported difficulties navigating the system, suggesting the need for alternative support strategies, such as targeted patient education or hybrid digital-human triage models. Timeliness and responsiveness were also critical themes, with many patients noting efficient handling of urgent issues, while others expressed concerns over delays or lack of follow-up [41].

The implementation of total triage at H&B reflects a similar commitment to digital transformation, as seen at the BUV Medical Center. However, while the BUV Medical Center serves as a single-site case study, H&B's adoption across all its general practices indicates a broader organizational initiative, providing insights into system-wide scalability and standardization. Both examples underscore the effectiveness of Accurx in optimizing call handling, improving patient access, and enhancing operational efficiency. Unlike the BUV Medical Center's focus primarily on general practice, H&B's implementation aligns with a growing trend of expanding digital triage platforms such as Accurx into secondary care settings, such as hospitals. This broader application illustrates the platform’s versatility and its potential to improve outpatient care delivery, reduce consultation wait times, and streamline clinical workflows beyond primary care.

The authors believe that this study is the first to evaluate the impact of a total triage platform on call wait times, providing valuable insights into its potential to improve operational efficiency and patient satisfaction. This innovative approach clearly demonstrates not only how digital tools can enhance healthcare delivery by reducing wait times and optimizing resource allocation but also introduces a new dimension to care navigation by capturing processes through digital datasets. This transition from traditional telephone consultations, typically managed by reception teams or care navigators, enhances efficiency through digital documentation and prioritization, streamlining patient interactions, and enabling better workflow management, ultimately supporting the evolving demands of modern healthcare delivery. Future studies should incorporate clinical outcome measures, track patient pathways more comprehensively, and analyze whether digital triage disproportionately affects certain patient groups, particularly older adults, non-English speakers, and individuals with limited digital literacy or access. 

Limitations

This study's findings are primarily limited to a single medical center, restricting the generalizability of results to broader healthcare settings. While Accurx has since been implemented at two additional H&B Medical Centers, comparative data from these sites were not included, preventing an assessment of variations in implementation effectiveness and patient outcomes. Expanding future research to multiple sites would provide a more comprehensive evaluation of Accurx’s scalability, adaptability, and long-term impact.

Another limitation was the lack of site-specific telephone data before August 2023, which hindered pre-implementation comparisons. Prior to the introduction of site-specific reporting, telephone data were aggregated across all H&B Medical Centers, making it difficult to isolate trends at the BUV Medical Center. As a result, evaluations of call efficiency and patient experience before Accurx’s deployment relied solely on available pre-implementation study data. Standardized data collection and site-specific reporting in future studies would enable more robust longitudinal analyses.

To strengthen future research, a multi-site study with standardized outcome measures would offer stronger evidence of Accurx’s impact. Ensuring comprehensive pre-implementation data across all sites would facilitate accurate comparisons of patient access, call efficiency, and system performance, enhancing the validity of findings and supporting broader conclusions on the effectiveness of digital triage in healthcare delivery.

## Conclusions

Integrating total triage with tools such as Accurx has improved healthcare delivery by reducing inefficiencies, optimizing resource utilization, and enhancing patient-centered care. While primarily implemented in primary care, its scalability suggests broader applications across specialties, with evidence of benefits in fields such as nephrology. However, challenges such as staff training and equitable access must be addressed to maximize its impact. Ongoing evaluation of clinical outcomes and patient satisfaction will be essential to refining these systems, ensuring that they continue to enhance efficiency, accessibility, and quality in healthcare delivery.
